# In-Situ Observation of Fracture Behavior of Ti-Aluminide Multi-Layered Composites Produced by a Hybrid Sintering Process

**DOI:** 10.3390/ma12091568

**Published:** 2019-05-13

**Authors:** Xiong Wan, Kai Zhu, Yanjin Xu, Baoshuai Han, Tao Jing

**Affiliations:** 1 Key Laboratory for Advanced Materials Processing Technology, Ministry of Education, School of Materials Science and Engineering, Tsinghua University, Beijing 100084, China; wanx15@outlook.com; 2 State Key Laboratory of Non-ferrous Metals and Processes, General Research Institute for Nonferrous Metals, Beijing 100088, China; zhu_thirteen@foxmail.com; 3 AVIC Manufacturing Technology Institute, Beijing 100024, China; hbshit@126.com

**Keywords:** multi-layered composites, hybrid fabrication procedure, in-situ observation, fracture mechanism

## Abstract

The fabrication of Ti-aluminide multi-layered composites have attracted great attention for their excellent mechanical properties, such as high specific strength, high specific stiffness, tolerable toughness, and low density. The preparation of the composite produced by a hybrid procedure composed of Vacuum Hot Pressing (VHP) and Hot Isostatic Pressing (HIP) using Ti foils and Al foils has been performed. Further, X-Ray Diffraction (XRD) and Scanning Electron Microscopy (SEM) equipped with Energy Dispersive X-ray Spectrometry (EDXS) were carried out to identify the microstructure and phase formation of the composite. In addition, an in-situ three-point bending test was conducted on the notched specimen to observe the crack propagation behavior carefully. The results indicate that the densified composite was obtained without any apparent voids and pores which could undesirably develop into the source of cracks. Furthermore, all the pure Al foils were totally consumed to form a series of the Ti-Al compounds through the diffusive reaction between the adjacent Ti and Al foils. Moreover, the in-situ observation demonstrates the initiation and propagation of cracks in the intermetallic layers and the role of residual Ti layers to blunt and bridge the cracks by their plastic deformation. This study provides a new strategy for fabricating the Ti-aluminide multi-layered composites.

## 1. Introduction

Advanced materials are critical for scientific and technological progress, as well as for modern industrial development. During the past decade, scientists and engineers have conducted lots of work to develop and fabricate a series of new materials that are much stiffer, stronger, and lighter than the traditional materials, in order to satisfy crucial and specific service requirements [1,2,3,4,5,6]. Especially in recent years, studies on the microstructures and mechanical properties of some rigid biological materials such as abalone and clam shells have shown that the intricately hierarchical microstructure of their shells is the essential reason why these shells exhibit excellent properties when undergoing an external load [7,8]. Noticeably, this unique hierarchical microstructure is over various scales and ultimately shown as a group of alternatively stacked brittle calcium compounds layers and ductile organic layers. The brittle phases supply the shells with enough strength, and the ductile phases play a vital role as glue to bond the contiguous brittle phase to form an assembly at last. Hence, this multi-layered alternating structure is referenced to do some innovative designations for fabricating materials with specific functional and mechanical properties [9].

It is well known that lots of studies have been conducted to develop new structural materials with multi-layered configuration like that of the aforementioned mollusk shells based on bonding two or three different metal foils, such as Ni-intermetallic laminate composite [10,11,12], Ti-intermetallic laminate composite [13,14], and Cu-Al laminate composite [15]. Of these new composites, Ti-aluminide multi-layered composite is the most investigated and it has many outstanding qualities, such as high resistance to corrosion, high strength, and low density [16,17,18]. Hence, Ti-aluminide multi-layered composite is regarded as a promising material employed for the aerospace industry. Until now, some fabrication procedures for producing the Ti-aluminide multi-layered composite have been used, such as reactive foil sintering, hot-roll bonding coupling with subsequent heat treatment, and explosive welding followed by annealing [19,20]. Generally, a band of collective voids or pores located near the centerline area of the obtained intermetallic layer always takes place after the fabrication process mentioned above. These defects could effectively induce to generate some stress concentration area around them to act as the sources of cracks to deteriorate the mechanical properties of the samples while loaded externally. In addition, few in-situ observations have been conducted on characterizing the crack initiation and propagation processes during the loading process. It has been proven that in-situ scanning electron microscopy (SEM) is a reliable and valid method that can clearly and timely record some valuable details of the damage process of the specimen [20,21].

Based on the above discussion, to obtain a dense multi-layered composite, a novel hybrid fabrication procedure composed of Vacuum Hot Pressure (VHP) and Hot Isostatic Pressure (HIP) was adopted to fabricate this bio-inspired multi-layered material by using the pure Ti and pure Al foils. Furthermore, the fracture mechanism and crack propagating behavior of this material were characterized and investigated by three-point bending test coupling with in-situ SEM.

## 2. Materials and Methods

### 2.1. Materials and Specimens Preparation

**Figure 1:**
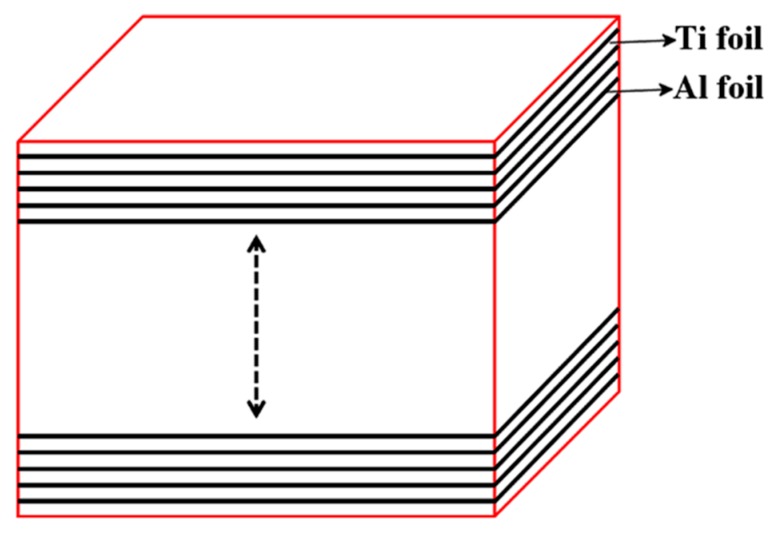
Schematic sequence of stacking of the elemental components.

In our research, the commercial Ti foils (purity ≥ 99%, BAO TI Group Co., Ltd., Baoji, China) and Al foils (purity ≥ 99%) with corresponding thicknesses of 100 μm and 40 μm were adopted. To improve the bond quality of the adjacent foils, the contaminants and oxidation layers on the surfaces of the Ti foils and Al foils were respectively cleaned by the 15% HF solution and 20% NaOH solution (more details can be found in [22]). Finally, the cleaned foils were sandwiched following the stacking sequence, as can be seen in Figure 1.

**Figure 2:**
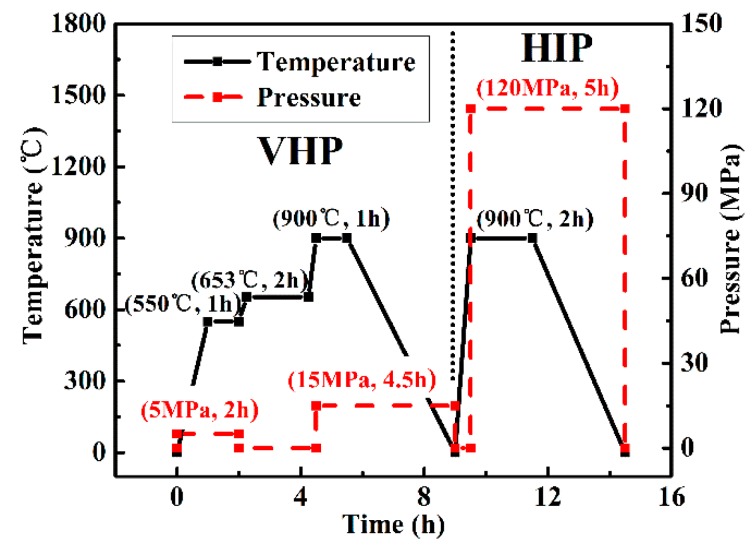
Schematic illustration of the hybrid processing technology.

Afterwards, the precursor was moved into the hot pressing furnace (ZT-40-20Y, Shanghai Chenhua Science Technology Crop., Ltd., Shanghai, China); the sintering parameters of this process are schematically shown in Figure 2. Firstly, to obtain a prelaminar contact between adjacent foils, we increased the temperature to 550 °C from the room temperature and kept this temperature for 1 h under a low pressure of 5 MPa. After that, the temperature rose to 653 °C while the pressure was decreased to atmospheric. This step was kept for 2 h to obtain a sufficient reaction and avoid forcing out any molten Al. Subsequently, the temperature was turned up to 900 °C and then cooled down. During this step, the pressure was increased to 15 MPa for compressing the composite. Furthermore, the Hot Isostatic Pressing (HIP) was chosen as to densify the composite. During this step, the sintered sample was encapsulated into an evacuated glass can (5 × 10^−4^ Pa) and then densified at 900 °C for 2 h, while the applied pressure was 120 MPa.

After sintering, the specimen was obtained from the fabricated composite by the electro-discharge machining technology and then inlaid in epoxy resin to form an assembly. Subsequently, the assembly was ground with metallographic abrasive paper and then polished with the diamond suspensions (6 μm, 3 μm, 1 μm, and 0.25 μm). To solve the problem of the work-hardening resulting from conventional grinding, a chemo-mechanical polishing using Al_2_O_3_ suspension (particle size: 0.04 μm) was performed. Scanning Electron Microscopy (SEM, Shimadzu Corporation, Kyoto, Japan) equipped with Energy Dispersive X-ray Spectrometry (EDXS, X-Max^N^ OXFORD INSTRUMENT, Oxford, UK) was used to examine the microstructures and phase composition. At the same time, phase identification was performed by X-Ray Diffraction (XRD, X’PERT PRO MPD, PANalytical B.V., Almero, the Netherlands). 

### 2.2. In-Situ Three-Point Bending Test

**Figure 3:**
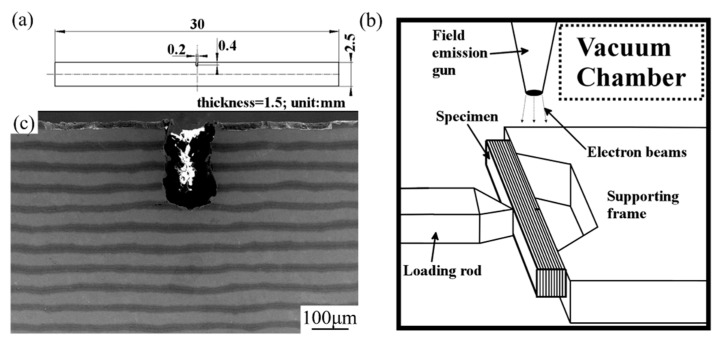
(**a**) Dimensions of the examined sample, (**b**) schematic illustration of the experimental stage in a vacuum chamber, and (**c**) initial morphologies of the area near the notch of the sample.

A servo-hydraulic loading system, installed in the vacuum chamber with a scanning electron microscope (Shimadzu Corporation), was used to conduct the in-situ three-point bending test with the constant loading speed of 0.1 mm·min^−1^. In order to record in-situ the crack propagation behavior, the test was interrupted intermittently to capture the in-situ propagation behavior of cracks. Then, the load procedure was resumed until the final rupture of the specimen. Figure 3 demonstrates schematically the dimension of the tested specimen and loading state in the chamber of the SEM, as well as the supporting distance adopted in this experiment is 20 mm. Herein, it should be noted that the orientation of the notch in the specimen is perpendicular to that of the layer and this orientation of the notch is named crack arrester orientation in some other researches [14,23]. Figure 3c shows the micromorphology of the notched specimen, and none of the cracks can be detected within the sample.

## 3. Results and Discussion

### 3.1. Microstructure Observation And Phase Identification

**Figure 4:**
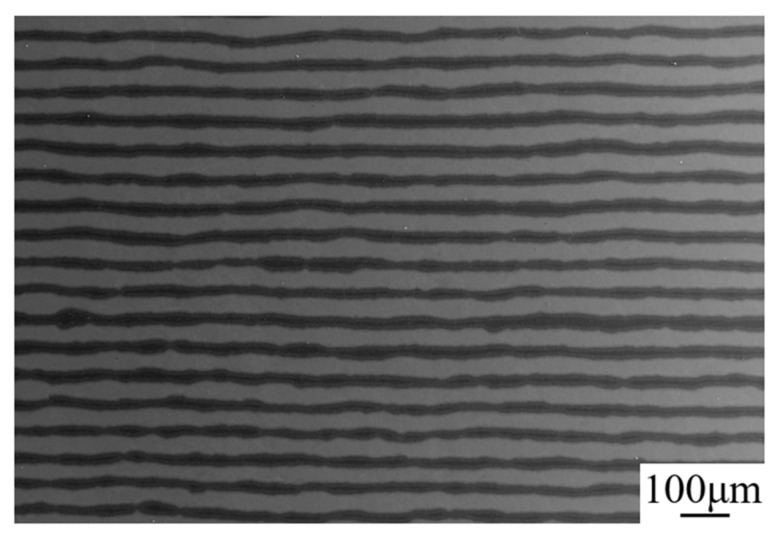
Typical microstructure of the as-received specimen.

A typical microstructure of this multi-layered composite surface is illustrated in Figure 4. It can be seen there is no defect like pores at the adjacent interface in this apparent multi-layered structured composite. As is mentioned above, even though the pressure applied during the VHP sintering process was not very enough to fully densify the sample, the super high compressive pressure resulted from HIP densifying process acted as a useful complementary step to crush the voids to enhance the density of the sample. Moreover, further observation indicated that the shapes of the alternating layers exhibited relatively straight at the macro level, but wavy at the micro level. This phenomenon may be ascribed to the distinguish reaction rates of spots between Ti and Al foils during the sintering process [18].

**Figure 5:**
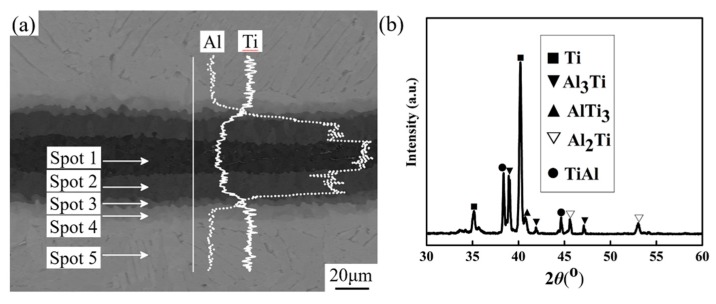
(**a**) Typical back-scattered electron (BSE) image of the Ti-Al reaction zone and Energy Dispersive X-ray Spectrometry (EDXS) linescan analysis results, (**b**) X-Ray Diffraction (XRD) pattern of this composite.

Furthermore, amplification characterization reveals there are four distinctive sub-structures distributed within the intermetallic layer, as can be seen in Figure 5a. To clarify the compositions and compounds of the intermetallic layer, EDXS and XRD were adopted. The concentration of Ti element evidently exhibited a graded distribution along the line crossing the adjacent layers and the concentration value of Ti element decreased progressively from the residual Ti layer to the center area of the intermetallic layer opposing to the distribution behavior of Al element. Subsequently, EDXS point-detection was adopted to identify the chemical compositions of each sub-structure of the intermetallic layer. The quantified results, as shown in Table 1 revealed that they are Al_3_Ti, Al_2_Ti, TiAl, Ti_3_Al, and Ti, labeled as spot 1, spot 2, spot 3, spot 4, and spot 5. Furthermore, XRD characterization demonstrates the above results, shown in Figure 5b. As mentioned by some previous papers [18,24,25], solid Ti and liquid Al had the abilities to react with each other to form some intermetallic compounds such as Al_3_Ti, Al_2_Ti, TiAl, Ti_2_Al_5_, and Ti_3_Al. Thermodynamic calculation on the Gibbs formation free-energy of these aluminides indicates that Al_3_Ti has relatively lower formation free-energy among the compounds mentioned above during the temperature range of 0–800 °C [26,27]. Although the Al_2_Ti and Ti_2_Al_5_ possess much lower formation free-energy than Al_3_Ti, some researches have been demonstrated that TiAl should be involved as the starting phase to form those two aluminides formation process [28]. Therefore, it can be deduced that Al_3_Ti is the first generated phase prior to other intermetallic phases due to its lowest formation free-energy at the interfacial temperature around 653 °C utilized in our research. After all of the liquid Al phase has been consumed into generating Al_3_Ti, in the following temperature post-process stage, aluminum atomics continuous to diffuse to the residual Ti layers from the Al_3_Ti compound due to the gradient concentration of the Al element to form Al_2_Ti, TiAl, and Ti_3_Al from the resultant Al_3_Ti layer to residual Ti layer [24,29].

### 3.2. In-Situ Three-Point Bending Test Observation and Detailed Fracture Behavior

**Figure 6:**
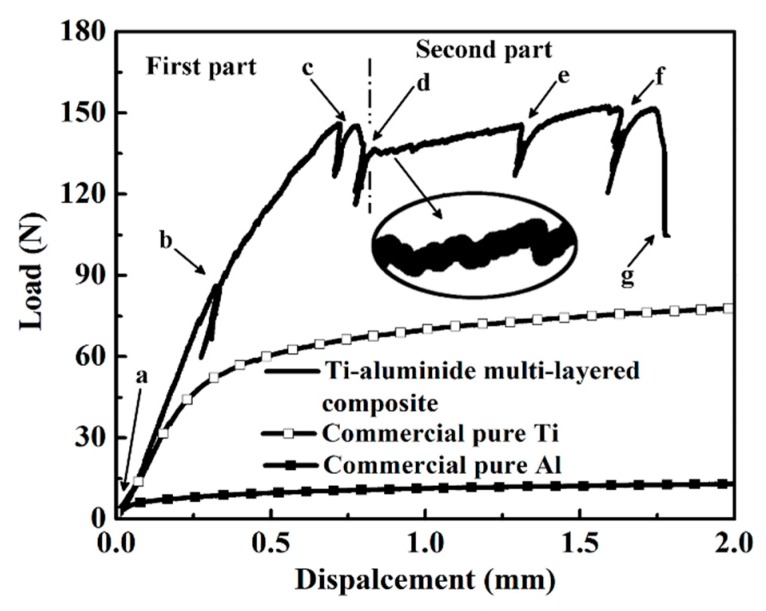
Load-displacement curve of the in-situ three-point bending test.

Figure 6 presents the typical load-displacement curve. It should be pointed out that the aslant tails of the line represent that several interruptions were performed during the mechanical test to capture the constantly changing morphology of the specimen before its catastrophic failure [21,30]. Through analyzing this curve of mechanical response, we divided this curve into two parts using the dotted line shown in the Figure 6: (1) the line-tendency goes up from the beginning and reaches the area near the interruption point “d”; (2) the line-tendency shows a drop followed by the first part and then goes up again before the final fracture. Moreover, lots of micro-fluctuations can be observed by amplifying the curve marked by an elliptical circle in Figure 6, which are resulted from the formation and propagation of the micro-cracks within the sample undergoing the external load. 

For comparing the mechanical responses between this composite and its components, the same testing procedures were conducted on commercial pure Ti and pure Al specimens and the results are illustrated in Figure 6. It is clear that the strength of the multi-layered composite is much higher than those of Ti and Al, while the ductility of the composite is lower than those of Ti and Al (typical pure metallic behavior with a long plateau after the yielding point).

**Figure 7:**
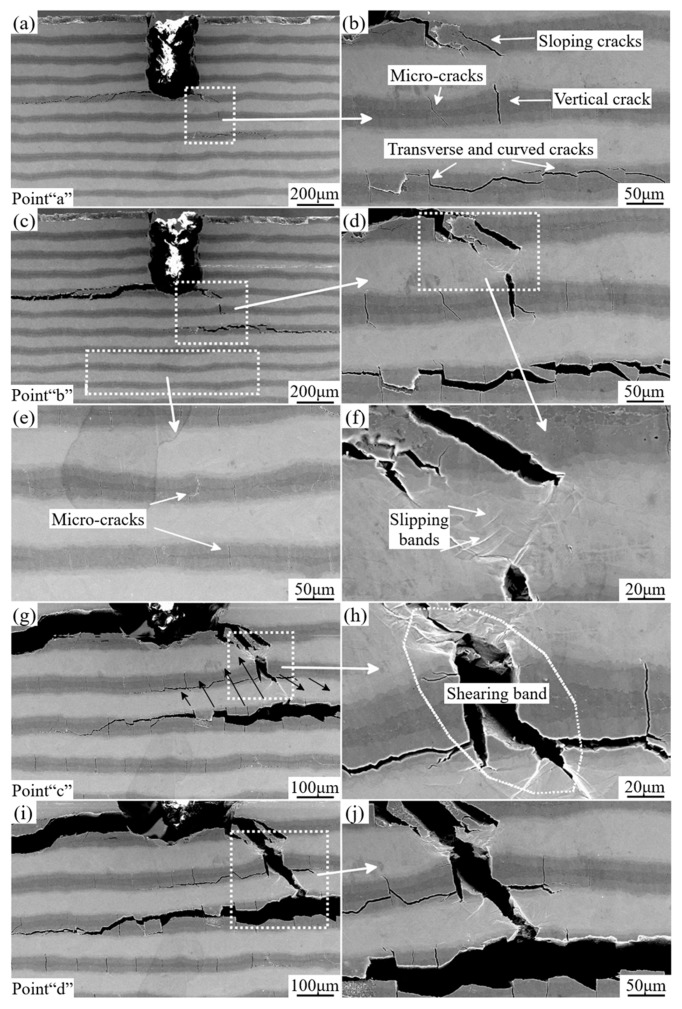
Scanning Electron Microscopy (SEM) micrographs of the in-situ three-point bending test corresponding to the first part of the Load-displacement curve representing the cracks nucleation and propagation process: (**a**,**b**) represent the detailed deformation morphologies of the specimen corresponding to the ”Point a” in Figure 6, showing several cracks initiated and propagated within the brittle intermetallic layers; (**c**–**f**) correspond to “Point b” in Figure 6, showing the crack bridging, slipping bands of Ti layers and more micro-cracks in intermetallic layers; (**g**,**h**) correspond to “Point c” in Figure 6, showing the shearing band in Ti layers and thorough fracture of the intermetallic layers; (**i**,**j**) correspond to “Point d” in Figure 6, showing the thorough fracture of the local Ti layers and Intermetallic layers.

Figure 7a–j shows the detailed deformation morphologies corresponding to some interruption points indicated in Figure 6. Firstly, at the beginning of load (marked as “a” in Figure 6), several cracks have been initiated and propagated within the brittle intermetallic layers near the notch site, which are shown in Figure 7a,b. Especially shown in Figure 7b, it can be seen that there are three categories of cracks marked by white arrows in the amplified area. According to the orientations of these cracks, they are named sloping cracks, vertical cracks, and longitudinal cracks, respectively [31]. Furthermore, there have already been some vertical micro-cracks (also marked by white arrow) in this area. It is clearly demonstrated that the orientations of those initial micro-cracks are perpendicular to that of the layers. Nevertheless, hardly any deformation traces within the Ti layer can be found at this time, which is consistent with previous studies [23,32,33]. With the increasing load, the widths of previous cracks become larger than before coupling with some new cracks generation corresponding to the point “b” in Figure 6. Meanwhile, some ductile Ti layers closing to the notched area have been sheared to exhibit plastic deformation and the crack bridging of Ti layers can be seen in Figure 7d,f, which introduces the cracks in one intermetallic layer to the next intermetallic layer by intermediate Ti layer, prolonging the cracks greatly. In Figure 7d, slipping bands which are generated during the shear deformation of Ti layers are detected. Besides that, more and more micro-cracks (shown in Figure 7e) appeared in intermetallic layers due to the increasing external load. The cracks propagate along the layers within the intermetallic layer rather than penetrate into the ductile layer and pass through the specimen directly, resulting from the lower the interfacial bonding strength between intermetallic layers and ductile layers than the strength to tear the ductile Ti layers. Thus, the brittle intermetallic layer is thoroughly ruptured due to the excess of the tensile stress (marked by black arrows in Figure 7g) and this tensile stress also results in plastic deformation, giving rise to strain strengthening of the ductile Ti layer at the moment of point “c” in Figure 6. Subsequently, at the end of first part (marked by point “d”), the ductile Ti layers are totally sheared, and the lateral morphologies of the sample at this point are shown in Figure 7i,j. Following the fracture of Ti layers, the tendency of the curve has a slight drop at the end of the first part in Figure 6.

**Figure 8:**
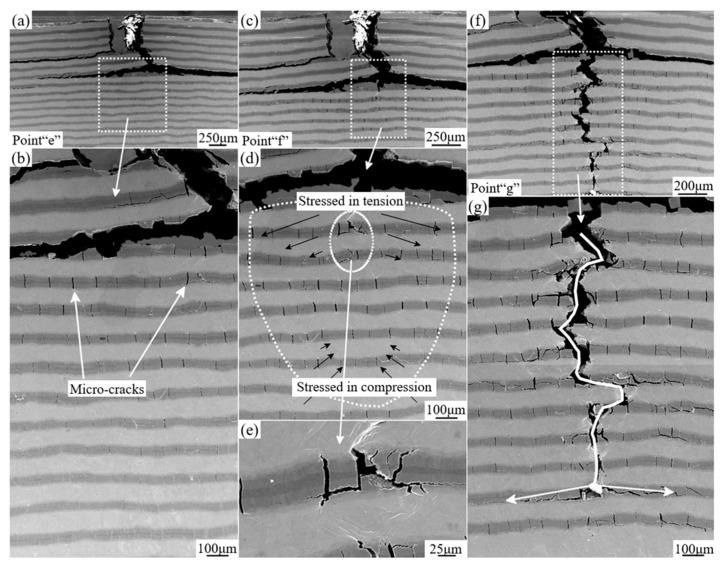
SEM micrographs of the in-situ three-point bending test corresponding to the second part of the Load-displacement curve representing the cracks propagation process and the final morphologies of the specimen after catastrophic fracture:(**a**,**b**) represents the detailed deformation morphologies of the specimen corresponding to the ”Point e” in Figure 6, showing lots of micro-cracks formed before the novel main crack exposure; (**c**–**e**) correspond to “Point f” in Figure 6, showing the distribution of micro-cracks with the mushroom shape; (**f**,**g**) correspond to “Point g” in Figure 6, showing the “zigzag” propagation of the main crack

Then, at the beginning of the second part, the tip of the main crack has been already deflected and blunted. Herein, additional energy should be provided for the continuous propagation of the crack. In Figure 8a, it can be found that lots of micro-cracks formed with the loading process before the novel main crack exposure. At the same time, the ductile Ti layer is gradually stretched to generate localized plastic deformation area, which is indicated in Figure 8d,e. Additionally, the distribution of micro-cracks within the plane exhibits mushroom shape (marked by white dotted loop) caused by the tensile stress condition [24,34]. Due to the above reasons, the crack density exhibits a graded distribution from the upside to the opposite side. Finally, with the continued loading process, the specimen is bent to be fractured. Here, it should be pointed out that the tip of the main crack is not straight, showing the “zigzag” propagation. And the tip of the main crack is often branched at the interfaces of Ti and Ti-Al intermetallic compounds, which is noted by white arrows in Figure 8g. Herein, based on the above observations, it can be shortly summarized that extrinsic toughness mechanism plays an important role, such as crack deflection and crack blunting et al [23,35]. 

Obviously, due to these mechanisms, the propagating paths of cracks are significantly changed and prolonged, enhancing energy absorption capacity of the composite [36].

## 4. Conclusions

In this work, we adopted a hybrid sintering process to fabricate the Ti-aluminide multi-layered composites, and analyzed the facture behavior with in-situ three-point bending test, the conclusions can be drawn as follows:

(1) The Ti-aluminide multi-layered composites were successfully fabricated from sintering an assembly comprising alternatively stacked pure Ti and pure Al foils by a hybrid procedure composed of Vacuum Hot Pressing (VHP) and Hot Isostatic Pressing (HIP).
(2) Microstructural observations indicated that all the pure Al foils were totally consumed to form a series of the Ti-Al compounds through the diffusive reaction between the adjacent Ti and Al foils, and none of the defects such as pores or voids were detected within the sample.
(3) The failure mechanism and crack propagation behavior of Ti-aluminide multi-layered composites were carefully observed by in-situ three-point bending test on the notched sample. Furthermore, the observation indicated that the cracks mainly initiate and propagate along the intermetallic layers. Moreover, residual Ti layers also play an important role to blunt and bridge the cracks by their plastic deformation at the tip of those cracks to arrest the cracks propagation and enhance the toughness of this composite.

## Figures and Tables

**Table 1:** EDXS points analysis shown in Figure 5.

Element	Spot 1 (at. %)	Spot 2 (at. %)	Spot 3 (at. %)	Spot 4 (at. %)	Spot 5 (at. %)
Ti	24.77	32.89	48.72	74.59	96.33
Al	75.23	67.11	51.28	25.41	3.67
Phase	Al_3_Ti	Al_2_Ti	TiAl	Ti_3_Al	Ti
